# Optimizing a knowledge translation intervention: a qualitative formative study to capture knowledge translation needs in nursing homes

**DOI:** 10.1186/s12912-021-00603-5

**Published:** 2021-06-21

**Authors:** Trine-Lise Dræge Steinskog, Oscar Tranvåg, Monica Wammen Nortvedt, Donna Ciliska, Birgitte Graverholt

**Affiliations:** 1 Western Norway University of Applied Sciences, P.O Box 7030, N-5020 Bergen, Norway; 2 Norwegian Research Centre for Women’s Health, Oslo University Hospital, Rikshospitalet, P.O. Box 4950, Nydalen 0424 Oslo, Norway; 3 McMaster University, 1280 Main St W, L8S 4L8 Hamilton, ON Canada; 4 VID Specialized University, P.O. Box 184, Vinderen, NO-0319 Oslo, Norway

**Keywords:** Professional nursing development, Evidence-based practice, Knowledge translation, Nursing home, MRC-framework, Complex intervention, Intervention development

## Abstract

**Background:**

Knowledge translation (KT) has emerged as an important consideration to reduce knowledge-to-practice gaps in healthcare settings. Research on KT approaches in nursing homes (NHs) is lacking. There is a need to understand the challenges faced in NHs and how these can be managed. This study is part of the larger IMPAKT (IMPlementation and Action for Knowledge Translation) study which addresses KT in NHs. The aim of the study presented here was to identify crucial staff and organizational needs in order to inform the development of a KT intervention in NHs.

**Methods:**

A multimethod qualitative approach was applied. We invited practice development nurses (PDNs) to describe current practice, and to identify problems and needs concerning KT in NHs. We followed the recommendations of the development phase of the MRC framework for developing complex interventions. Data were collected through four focus groups and participatory observations in six NHs. Analysis was conducted according to structural thematic analysis based on a phenomenological hermeneutic method.

**Results:**

We identified three themes that expressed the PDNs’ perceived needs for successful KT implementation: (1) narrowing the PDN role, (2) developing an EBP culture and (3) establishing collaborative alliances. Nine subthemes derived from the PDNs’ experiences and current practice, illustrating needs at individual, relational and organizational levels.

**Conclusions:**

Rigorous development of complex interventions may add relevance to the intervention, increase the likelihood of success and reduce research waste. Insight into the NH context and organization have helped us define problems and articulate needs that must be addressed when tailoring the IMPAKT intervention.

**Trial registration:**

The IMPAKT trial was retrospectively registered in the ISRCTN Registry (Trial ID: 12,437,773) on March 19th, 2020.

**Supplementary Information:**

The online version contains supplementary material available at 10.1186/s12912-021-00603-5.

## Background

The field of knowledge translation (KT) has emerged to bridge the *knowledge-to practice gap* in healthcare settings [1]. KT refers to the process of moving knowledge into healthcare practice and policy and includes the synthesis, dissemination, exchange, and application of knowledge to improve health and strengthen the healthcare system [2]. There has been an increasing amount of attention given to practice development and quality improvement within healthcare practice, moving to evidence-based practice [3, 4]. In nursing, practice decisions have commonly been based on intuition, practice experience and analytical reasoning [5]. Evidence-based practice (EBP) is an approach to clinical decision-making that incorporates a search for the best and latest evidence, clinical expertise, assessment, and patient preferences within a particular context [6]. Evidence-based practitioners need to understand the conceptual basis for KT as this is a crucial framework to introduce best evidence into practice [7]. Within the present project, implementation is understood as a step within EBP, acknowledging that implementation is a highly complex process. While there is considerable conceptual confusion and overlap in definitions and processes [8], the broader KT framework [9] was utilized to guide this project.

There have been a considerable number of primary research studies and systematic reviews examining KT interventions [10], but few have been related to the NH setting [11, 12]. This is a paradox given how the ageing population puts pressure on NHs [13–15]. This patient population has grown in almost all OECD (Organization for Economic Co-operation and Development) countries [16] and represents the highest growth in spending amongst healthcare sectors [14].

Competent staff supported by continuous professional development are the foundation for successful healthcare organizations [17]. The literature reveals that implementing EBP is complex since the majority of nurses worldwide state that they are familiar with, have positive attitudes towards, and believe in the value of EBP in improving care quality and patient outcomes. However, nurses perceive their own EBP knowledge and skills as insufficient for employing EBP and rarely use the best evidence in practice [17]. Barriers to change that can impair the effectiveness of interventions designed to improve professional behavior and practice [18] within NHs have been classified as organizational barriers [19–22], contextual barriers [23, 24], and individual barriers [17, 25].

Educational programs targeting EBP can no longer rely on passive education methods or knowledge diffusion but need to develop specific approaches to enhance KT in practice [26]. The updated “Framework for the Development and Evaluation of Complex Interventions” [26] produced by the UK Medical Research Council (MRC), provides guidance to enhance the uptake of interventions in practice through four nonlinear phases: Development, Feasibility and Piloting, Evaluation, and Implementation. The current study is aligned with the requests by the World Health Organization (WHO) to evaluate KT initiatives within the geriatric setting [10, 27]. Research on KT approaches in care for older adults is necessary to gain an increased understanding of the challenges related to KT in NHs and how these can be met [10, 11]. Interventions tailored to prospectively identified barriers are more likely to improve professional practice [28–30]. As such, context specific knowledge is crucial to enhance the likelihood of developing an intervention that will improve professional practice [29].

The aim of this study was to identify crucial staff and organizational needs in order to inform the development of a KT intervention in NHs.

## Methods

We applied a multi-method qualitative formative study design utilizing Lindseth and Nordberg’s [31] method for structural phenomenological hermeneutical analysis of life experience, inspired by Ricoeur [31, 32]. We focused on the multiple, collectively constructed realities accessible through the language, discourses, and organizational structures within the NH context. We found these lenses appropriate for observing, understanding, and reporting the KT needs of PDNs. The central proposition is that the professional in-depth perspective of PDNs must be understood from within rather than explained from an outside perspective. In other words, external descriptions and explanations alone are not sufficient to obtain an in-depth understanding of experiences related to human existence [31, 32]. Thus, we gained insight into their professional lifeworld and the underlying meaning of their experiences and perspectives. Ricoeur argues that when discourses are recorded in writing, it creates a distance that can release the meaning from the spoken narratives and can open up interpretation options. Through this approach, movement from a surface interpretation to an in-depth interpretation of the transcribed empirical material can be obtained [32].

### Setting

This study took place in an urban-suburban municipality in the western part of Norway. Municipalities in Norway are responsible for NH services [33]. At the onset of this study, this municipality had 23 public NHs.

### The IMPAKT study

The IMPlementation and Action for Knowledge Translation (IMPAKT) study consists of a collection of research enquiries related to KT in Norwegian NHs [34]. The overall aim of the IMPAKT study is to address the knowledge-to-action gap from the perspective of healthcare professionals in public NHs. By using an integrated KT (IKT) approach, end-users are involved in the process of developing and testing a KT intervention in a cluster randomized controlled trial. It is anticipated that the trial will consist of two components. The first component will be an educational KT program tailored to the needs of the NH staff and the organization. The second will be a facilitation-upon-implementation component to implement evidence-based recommendations for a collectively chosen area of practice.

The study presented here will help to identify participants and their needs to inform the curriculum of the educational KT program. The IMPAKT trial is described in more detail elsewhere [34, 35].

### Recruitment and characteristics of participants

We invited a strategic sample [36] consisting of PDNs in public NHs to participate in this study. PDNs are employed in NHs to drive quality improvement, including educational activities to promote professional development. Registered nurses (RNs) holding a bachelor’s degree can apply for this position. PDNs have a formalized job description from the municipal agency, and most of them have an additional clause in their contract requiring them to fulfill tasks specific to their particular NH, delegated by the NH director. Studies in hospital settings reveal that KT is one of the responsibilities of a PDN. PDNs act as bridge-builders between scientific knowledge and local professional training [37]. We found no other study of KT within the role of a PDN (or equivalent) in NHs. The PDNs were informed about the study during one of their regular meetings, then invited to attend focus group interviews. At the time of data collection, 18 of the 23 public NHs employed a PDN, 17 of these participated in this study (Table 1).

**Table 1:** Participant characteristics

Year in the position		Percentage of full-time equivalent as a practice development nurse		Age		Post graduate education	
< 1	3	20 %	3	< 40	4	None	2
1–5	7	40–80 %	6	40–50	6	Clinical certification	14
> 5	7	100 %	8	> 50	7	Master’s	1

### Data collection

The data were collected between May and November 2018. We carried out four focus group interviews with three to five PDNs in each. An interview guide was developed following Krueger and Casey’s recommendations [38] and contained 12 questions (see Additional file 1). We analyzed the interviews concurrently with the data collection to allow emerging themes to be incorporated into later interviews. The questions were open and explorative, inviting participants to describe their experiences of current practice, problems and needs related to EBP and implementation. We distributed the interview guide to encourage reflection before attending. Each interview lasted for two hours, including time for an introduction and final remarks. The co-moderator took notes on non-verbal communication, group dynamics and asked supplementary questions. After each interview, the moderators reflected upon the interview process and wrote a summary. In addition, participant observation was carried out; six of the participants agreed to have a researcher follow them during a usual workday. Collecting data from focus groups and from participatory observation is recommended in phenomenological hermeneutic studies [39]. This combination enabled us to examine our preliminary interpretations and observe dimensions not covered in the focus groups. Field notes were used as a narrative account of what happened [40].

### Data analysis

The audiotaped interviews were transcribed verbatim. The participants were anonymized, and the tapes were erased. Data were analyzed employing Lindseth and Nordberg’s [31] phenomenological hermeneutical method, following their three analytic steps. First, a *naïve reading* was done to grasp an overall impression of the text. The transcribed interviews and field notes were read several times to inductively gain access to the PDNs’ lived experience within their role, their descriptions of current practice, their perceived problems and needs regarding EBP and implementation. In this phase, we moved into the world of phenomenology. As opposed to bracketing personal experiences, expertise, and biases regarding the issues under study, we openly reflected on, shared, and attended to our subjectivity during collection and analysis of data, thus adhering to the hermeneutic tradition [31, 32]. The naïve understanding of the text revealed the direction for the *structural analysis*, which was the second step. At that point, the text was divided into meaning units that were condensed into subthemes and themes (Table 2).

**Table 2:** Example of link in the analysis process

Meaning Unit	Condensation	Subtheme	Theme
*…those who shout the loudest, those who are the hardest opponents − for you to change their minds… because they are often strong personalities in the wards… so say ; if you give them a task that they are passionate about… you have to know… spend some time to know your own organization*	It is important for the PDN to know the staff. There are benefits when working with the strong personalities to be on side with the PDN.	Mapping competence, and appointing collaborators	Establishing collaborative alliances

By using phenomenology, we provide an *explanation* of what the text says in a structural analysis of its parts. Thus, the objective of the structural analysis was to identify patterns of meaningful consistency and to seek explanations of the text. However, we also aimed for an *understanding of the text through interpretation* − moving beyond what the text said (*its sense*) to understand what it talked about (*its reference*) [32]. The objective of the structural analysis was therefore two-fold. The first objective was to identify patterns of meaningful consistency and seek explanations of the text. The second was to gain a deeper understanding of the PDNs’ perceptions through a continual interpretation of the parts of the text and the text as a whole, also called “the hermeneutic circle” [32, 42]. Member checking was conducted at this stage, in line with Korstjens and Moser’s recommendation [41]. We presented our preliminary analysis of the interviews and observations at a workshop to which all PDNs were invited. Participants were invited to provide feedback that gave supporting or contradictory perspectives, which added nuances to the analysis.

The third and last step was intended to develop a *comprehensive understanding (interpreted whole).* We aimed for a critical in-depth understanding of the text as a whole. Individually and collectively, we reflected upon and discussed our preunderstandings and our initial naïve understanding, and the later findings from the structural analysis [31, 32]. At this point, we moved from naïve readings into developing a theoretical understanding of the empirical data. For this, we added a deductive approach, utilizing Bleijenberg’s proposed elements for the development of interventions. This entails identifying and defining the problems that the intervention seeks to solve, determining the recipients’ and providers’ needs, and examining current practice and context [29]. The Standards for Reporting Qualitative Research, SRQR [43], was utilized to ensure that all qualitative research aspects had been reported (see Additional file 2).

## Results

We identified three themes and nine subthemes expressing the PDNs’ perceived needs for successful KT implementation derived from the PDNs’ experiences of implementing change (Table 3).

**Table 3:** Classification of themes and subthemes

*Themes*	*Subthemes*
Narrowing the PDN role	- Prioritizing quality improvement - Establishing authority - Gaining leadership commitment
Developing an EBP culture	- Creating a learning environment - Improving EBP competence and support - Having access to evidence-based guidelines and procedures - Finding relevance and usefulness in quality improvement efforts
Establishing collaborative alliances	- Mapping competencies and appointing collaborators - Cooperating with other PDNs

### Narrowing the PDN role

#### Prioritizing quality improvement

The PDNs found it challenging to explain their distinct role. They seemed to be involved in most processes within their NH facilities and felt responsible for the maintenance of daily operations. They expressed loyalty to their colleagues and a feeling of obligation to help them out in their busy everyday care for residents:

*Everyday life here is hectic; we don’t have people for all the tasks. I must take on duties that are not mine; who else will do it? We help to make everyday life work out best for everyone. … Being able to concentrate merely on quality improvement? No, that’s just not how it is…*.

During an observation, a PDN commented on the expectation from the management to show flexibility in her role. She opened the shared PDN job description while stating:

*I wish I had more explicit expectations from the municipal agency − what am I expected to do?*

The PDNs expressed frustration with the broad job description and the additional tasks delegated by the NH director, which resulted in unpredictable ad hoc duties. Responsibilities varied across NHs despite the same job description. Those with part-time positions found it challenging to meet the same expectations as full-time PDNs. Several addressed the importance of structuring their work to give priority to their primary task of quality improvement, as they experienced numerous requests from all levels of the organization:

*I think we need to be a bit inconsiderate and organize our schedule to prioritize what is essential to our work as PDNs … we must dare to say no to tasks that others assign us … but you must be pretty confident to do that.*

The PDNs often felt overwhelmed by the number of implementation projects being presented as equally important without a realistic time frame to deal with them properly:

*There are too many things going on at the same time. Somehow, everything is presented as equally important…. At times, you can’t cope… I may sound very negative, but quite honestly, it’s too much − everything is presented with the same importance. I wish we could stop and get projects implemented well before jumping to something new…before we get new instructions. I think we must calm down and maintain one focus for a longer time.*

Work accumulated as the number of projects continuously increased without any being removed. The PDNs expressed a need for clearer priorities and opportunities to focus on quality improvement.

#### Establishing authority

Some participants perceived an attitude among colleagues signaling that PDNs’ primary stated work was not recognized. During an observation, a PDN pointed at her mailbox where there was a pile of letters and brochures:

*Look*. *Whenever they don’t know where to put a letter, it ends up on my shelf; that’s how it is around here!*

The ambiguous PDN role seemed to mirror the unclear expectation of the PDN. This lack of clarity was true for staff as well as for the management. Several participants underlined the importance of having the opportunity to attend meetings where important decisions were made. They attended management meetings where they had the option to address current issues and influence areas of focus, yet none was involved in long-term planning of quality improvement. Some said that they lacked authority and power to influence quality improvement and wanted a stronger voice, claiming that the management did not recognize the PDNs’ responsibilities:

*Matters of quality improvement aren’t properly rooted from the top (management). We need to make this (quality improvement) as important as other issues. If we only had a structured long-term plan, which we could then refer to … My job is challenging because I have no authority. I am more like a supporter − you must invite yourself in*… *I would have liked to have a more significant role in the management group’s plans for quality of care.*

The PDNs expressed a need to clarify their responsibilities and expectations. They believed this would give greater authority to their role and increase the possibility of prioritizing EBP and quality improvement.

#### Gaining leadership commitment

In all focus groups, support from different leadership levels was emphasized as essential to the success of any implementation project. Participants described the head nurses as driving forces for implementation:

*…to get things implemented, we are dependent upon efficient head nurses to take responsibility. … They are in the wards, attending important meetings… to engage them to take an active role in implementation projects and take leadership… it (the implementation) is dependent on it… I can’t be in every single morning meeting, so if the head nurses don’t take leadership in implementation projects, then it won’t work.*

The PDNs emphasized how they were also dependent on the director’s commitment, first by providing authority to implementation projects, and second by allowing the PDNs to downgrade some tasks to create room for working with quality improvement.

### Developing an EBP culture

#### Creating a learning environment

All PDNs expressed an explicit responsibility for facilitating and teaching in their respective NHs. However, they articulated that poor information flow, low attendance, and certain characteristics of the context compromised their ability to fulfill their responsibilities. They claimed that the NH staff rarely opened their work e-mails, and many never used a computer at work. This made it difficult to reach out to these colleagues. Consequently, delivering teaching sessions was unreasonably time-consuming. Organizational challenges, great variation in staff competencies, and lack of a learning culture were all challenging factors, as expressed by this PDN:

*Teaching is difficult. We have registered nurses, licensed practice nurses, care aides, and students. How do you reach them all?*

The PDNs pointed out that students and aides were particularly important to target, as they often made up the majority of staff on shifts during the vulnerable hours of evenings, nights and weekends. Several examples of a poor learning culture were given. In many places, the PDNs’ sessions were not accepted as legitimate absences from the wards. Additionally, the PDNs expressed concern that staff were reluctant to reveal their knowledge gaps because they feared that they did not hold the level of competency expected of them:

*I think that when I ask… everyone is not completely honest about their proficiency levels and where they lack abilities*.

One PDN was concerned that NH staff were trapped in a state of performing unconsidered routines in their everyday work, without critical reflection. This was especially true amongst longer-tenured staff:

*… they know exactly what to do when they come to work… they claim that this has worked for 20 years, and it will work just fine for 20 more! They even say right out that, “no, we’re not doing it, we’ve tried that before and it doesn’t work"… they simply refuse to do it!*

The PDNs emphasized the importance of motivating the staff to appreciate how new knowledge can improve quality of care and can ensure that work is not a mere routine. Several PDNs shared their frustration at failing to evoke the interest of their staff and finding that too many projects implemented at the same time led to resistance and rigidity from the staff.

#### Improving EBP competence and support

Several participants found it hard to explain the essence of EBP. One claimed that EBP had become a buzzword without much content, and that staff had falsely convinced themselves that their practice is based on research. This has produced a disparity between actual versus expected knowledge and understanding:

*It has become a blurry term. It’s an expectation, something we have to do, base our work on evidence. Still, I think people fail to grasp the notion of what evidence-based practice is all about… It’s challenging not knowing aspects of my job that I am expected to perform.*

There were no requirements for formal education in EBP or experience with implementation to qualify for the job as a PDN, but the participants felt EBP, and implementation were essential parts of their responsibility. One of their tasks was to be a resource for nursing students having their practical placement. A PDN expressed a feeling of lagging behind and addressed a need for support:

*They (students) are more updated on recent research… I find it embarrassing. They may have learned something in school, and we do what we learned… but how do we find what is best? As a professional, it’s an awkward position to be in when I don’t even know where to search for the best information.*

The PDNs acknowledged the need to be updated on research findings as they recognized that their duties and responsibilities in NHs had become more complex, with a notable shift in tasks from tertiary care to municipal care:

*We have become more and more like mini hospitals… We treat a lot more conditions in NHs today than before. It is desirable that competence should be increased correspondingly… I do think it (EBP) is useful for us as PDNs that we gain competence and bring EBP into focus. I believe that it is useful for everyone, so it’s our job to convey its importance… to leaders, head nurses, and in the wards. I believe that once you know what it is, you realize the benefits, and then you acknowledge the need for it.*

Competence in teaching, mentoring, and implementation was self-taught for the majority of participants. They all agreed that they could benefit from courses and support for EBP, and from learning more about implementation strategies.

#### Having access to evidence-based guidelines and procedures

 Most PDNs preferred to receive ready-to-implement procedures and guidelines and trusted the quality improvement manager at the municipal agency to ensure that these were evidence-based. However, some PDNs expressed frustration and feared that procedures were distributed without any thought for quality or proper implementation:

*I think that the municipal agency needs to be more involved to facilitate us in becoming an organization that utilizes evidence-based practice … not just say that we do it…*.

Occasionally, PDNs found themselves trapped, uncertain whether they could trust the procedures from the municipal agency:

*I struggle between wanting to check where their procedures come from, what it is based on … and not having time to search for updates … so I have to trust that they have based it on the best available knowledge. But I can’t be sure. I think that… as PDNs, we need to ask more critical questions… dare to be confident and critical… I would like that…*.

Some PDNs stated that their motivation to implement a procedure was lower when they knew it was not based on the latest research. They suggested a system that would ensure quality before proceeding to implementation. Moreover, they called for a standard evidence-based procedure manual and an opportunity to have access to tools supporting EBP.

#### Finding relevance and usefulness of quality improvement efforts

The PDNs highlighted the need to tailor implementation efforts to the specific NH context. Most of them valued initial collaborative meetings with staff, perceiving that these meetings enhanced staff participation and ownership of the implementation project, as well as increasing chances of uptake and lasting changes:

*If staff feel that changes are forced upon them, then it’s impossible. If you manage to demonstrate relevance and usefulness for them and our patients, then you create interest, and that’s a crucial accomplishment to succeed.*

Several PDNs shared positive experiences from previously successful implementations. They highlighted the need to prepare and create thorough implementation and evaluation plans. They expressed the need to avoid quick solutions and underlined the importance of providing enough time to achieve successful implementation. They had experienced rapid change provoking resistance and recommended taking a slow pace, acknowledging that making a change in NHs takes time.

### Establishing collaborative alliances

#### Mapping competencies and appointing collaborators

The PDNs stressed the importance of knowing the staff well enough to map their competencies. They shared the perception of having a unique overview of the institution, the wards, and the employees. They introduced, coordinated, and facilitated implementation projects, but success was dependent on close relations with the staff:

*You have to be smart and choose your alliances and partners…it is not possible to achieve changes all by yourself…*.

The PDNs identified staff who could act as supervisors for others or be “super users” for certain clinical matters. This assisted accountability in implementation processes. Some PDNs emphasized the importance of confronting their opponents, working to get them on their side, as they were often opinion leaders (see example in Table 2).

#### Cooperating with other PDNs

The municipal agency organized regular meetings with the PDNs to provide information and to nurture the PDN network. All participants appreciated having this opportunity to cooperate and share knowledge and called for further collaboration on tasks of common interest. The participants valued productive interactions and relations with PDNs in other NHs, expressing this as a prerequisite for developing EBP in their own NHs:

*I wish this network of PDNs could be more structured… and that we had the opportunity to raise more issues that we are struggling with and get input and feedback from each other. I wish they were clearer on how research had informed what they asked us to implement; that’s the kind of input we want.*

PDNs who were new to their position felt that the PDN forum did not fulfill its potential to work strategically on implementing the latest research evidence. In their view, this forum could improve and increase their opportunities to become better PDNs:

*Working more collaboratively with this group could have helped me in my job. Most of the time, I work alone, and I often struggle with issues that would be useful to discuss with someone.*

Several PDNs suggested more structured meetings with a focus on presenting and discussing current practice, research, evidence-based knowledge, and implementation.

## Discussion

This study contributes to the identification of staff and organizational needs that should be considered when designing KT interventions in NHs. The perspectives were gathered from PDNs who were responsible for quality improvement and professional development in their NHs. The PDNs portrayed a fragmented system and revealed needs at a range of interwoven levels, including individual, relational and organizational levels. At the individual level, a root cause to the many challenges that PDNs identified was highly conflicting expectations. This was clearly illustrated by their formal job description together with stories and observations of a typical workday. Similar findings have been previously reported. For instance, Dwyer [44] found that nurses felt unprepared for the escalating complex role they held in NHs; this reflected a lack of clarity of expectations and training. Others have pointed to the absence of precision and consistency in advanced nursing roles and have described how these become barriers to quality improvement [44–46].

The policies directed at improving the quality of care and KT in long-term care are numerous and come from multiple levels [47]. National policies in Norway mirror the expectations of WHO [27]. Leaders of patient care are accountable for ensuring that clinical care is evaluated against best-practice standards and adjusted whenever necessary [47]. PDNs are in a key position to accommodate these legislative demands, but, as found in this study, they are neither properly prepared nor given the status, time, or room to perform this role. In a large integrative review of 37 studies, Saunders et al. [17] found similar results across a range of clinical settings. Saunders et al. suggest that nurses self-report insufficient competence to utilize EBP. An imbalance between expected and actual clinical competencies was shown in a Norwegian study [22], implying that NHs may fall short of professional and societal expectations.

Many of the known barriers to EBP, including time, resources, and competence to perform the steps of EBP, are evident in the NH setting [23, 48]. This group of PDNs questioned whether their job was to search for and develop standards of care or to facilitate the implementation of best practice standards. This mirrors the question posed by Strauss et al. [49] as to who are doers, users, and replicators of EBP. The PDNs in our study raised issues related to differentiation of roles within EBP and were particularly concerned with the lack of attention given to the implementation of new knowledge. They expressed a need for tools, training, and opportunities to plan, adapt, facilitate, and evaluate implementation projects. They also emphasized a need for ready-to-implement evidence-based guidelines and procedures and questioned who held this responsibility. According to the PDNs, implementing new knowledge is currently superficial, since new practice changes are constantly introduced, often without a transparent evidence base. Most PDNs reported that the combination of insufficient training on implementation, with feelings of being overwhelmed, resulted in poor implementation of many projects. As part of the solution, the PDNs emphasized that leadership support was needed to build a culture based on EBP. Previous research has extensively shown that leadership commitment is a prerequisite for optimizing quality improvement [50, 51], and that without it, achieving change is unlikely to happen.

### Implications for the IMPAKT intervention

This study has helped us to gain a better understanding of PDN needs that should be taken into consideration in the development of the IMPAKT intervention. By examining current practice and provider preferences and capacities, we achieved a clearer picture of the problems, thus informing the intervention design regarding the timing, content, intensity, dose, and information of “who, how, and what” [29]. Our preunderstanding that PDNs play a key role in driving quality work in NHs was confirmed. Consequently, PDNs will be the main target group for the educational component of the IMPAKT intervention. This first component of the intervention will need to consider identified learning needs in leading change and KT efforts. An educational KT program in leading change needs to build on what the target group knows, or lacks, beforehand. In this case, the PDNs appeared to have positive attitudes towards EBP and KT, but disclosed gaps in basic EBP competencies, such as searching and critical appraisal skills. The educational KT program was therefore tailored according to their needs [34, 35].

Building competence amongst individual intervention providers is crucial, however, to embed new knowledge in the implementation context, the PDNs are dependent on support at the organizational level. When it comes to developing competence, the focus and responsibility are often placed on professional practitioners, and one risks ignoring the responsibilities of the organizational level [52]. The PDNs perceived the context more as a task-oriented operation rather than having a culture characterized by ongoing learning and development. In order for IMPAKT and other quality improvement projects to make a difference, it is paramount for the organization to recognize that they are a learning organization. Learning in the context of care facilities is affected by contradictions: the organization’s need for effectiveness on the one hand and the need for professional development of staff on the other. Time spent on learning is often perceived as an additional burden that disrupts the work. The management of healthcare organizations needs to be aware of the significance and value of learning from the perspective of their organization’s results, and to emphasize learning in their strategic plans [53]. New knowledge and competence need to be embodied in the organization itself, and often this means that structural changes and adjustments are required [54]. Accordingly, the municipal agency, as well as the NH director, must acknowledge their responsibility to facilitate opportunities in order for organizational change in EBP cultures to develop. Several healthcare establishments have declared themselves to be a learning organization (LO) in order to construct a culture of maintaining professional competence and adapting to changes [55, 56]. Previously a Canadian study found nurses participating in LO initiatives improved their skills related to knowledge translation, communication, collaboration, timesaving, and standardizing practice. They also experienced a stronger sense of autonomy and recognition from other NH staff [57]. This is in accordance with the situation desired by the PDNs in this present study: establishing authority, having opportunities and tools to advance their own professional competencies as well as contributing to the development of a culture where they could facilitate learning and promote EBP. One aim of an LO is to establish a shared common vision [54]. In achieving this, staff perform tasks because they want to, instead of being told to do so. The PDNs emphasized the relational aspects of their role and expressed a need for mapping competence in the organization to efficiently coordinate and collaborate with their staff. Sound relations between staff and the ability to cooperate in utilizing knowledge and expertise in the working community create a positive learning culture, which affects the quality of care and practice development [52]. In an LO, activities are seen as having an impact beyond the individual, as an LO includes the NH wards, the relational staff interactions, and the networks outside the organization [53].

The PDNs valued the establishment of network meetings among the PDNs, arguing that the network promoted collaborative alliances and positive peer relations across NHs. This finding has previously been described by McGilton et al. [20]. The PDNs described the importance of being part of a collaborative learning network. Consequently, we suggest that the PDNs meet regularly throughout the implementation period.

Limitations of this study include the fact that only PDNs were invited to be participants. However, being appointed responsible for EBP and quality improvement projects in their respective NH, PDNs were central to inform the IMPAKT intervention, as well as to facilitate it. The purpose was to investigate the KT needs of PDNs. However, the term knowledge translation (KT) was unfamiliar to the participants and therefore the terms *EBP* and *implementation* were used in the interviews. A strength of the study was the high participation level, 17 of the 18 eligible PDNs agreed to participate. Traditional descriptive methodologies to understand complex healthcare interventions have often fallen short of comprehensively explaining intervention efforts [58]. To avoid this, we used a phenomenological hermeneutical approach, which we found beneficial for illuminating the PDNs’ in-depth perceptions of crucial staff and organizational needs for KT to be successful in NHs. In focus groups, it can be a challenge to obtain nuanced, in-depth perspectives [39]. This was countered by combining the focus group interviews with participatory observation. This enabled us to investigate how participants expressed themselves in bodily terms, how they interacted with others, as well as how they performed in their role as a PDN in everyday practice. This combination, we believe, gave us richer data, where statements were substantiated by behavior and vice versa [39].

To enhance study trustworthiness, we aimed to strengthen research credibility, dependability, confirmability, and transferability [59]. Credibility was sought by performing investigator triangulation with three researchers reading the interviews and observation notes and thoroughly discussing and reflecting upon the entire analytical process. A member check [41] was organized in a workshop. PDNs met to discuss ways of interpreting the data in order to increase confidence in the preliminary findings. Dependability and confirmability were sought by transparency in data collection using an audit trail [41] containing summaries and reflections of interviews and observations that were discussed within our research team. To increase transferability, we collected sufficient data to provide a detailed description of the PDNs’ experiences, illustrated by a number of direct quotes and observations [38, 59]. Finally, we emphasized communicating the findings in an everyday language to reflect the PDNs’ expressed needs as closely as possible. There was substantial agreement among the PDNs, implying that findings may be transferable to similar NH contexts. Ricoeur [60] underlines that transferability and reliability lie in recognition of others. Accordingly, we believe that knowledge gained from this study can be applied to other contexts as a basis for discussions when planning KT interventions. Details of the development process of interventions (in this case the IMPAKT intervention) are provided to enable others to make links between intervention development endeavors and the result of the intervention [61] and allow replication [29].

## Conclusions

Rigorous development of complex interventions may add relevance to end-users, increase the likelihood of effectiveness of the intervention and reduce research waste. This study was undertaken to support the development of an IKT intervention. Our multi-method qualitative approach identified PDNs as the key target group for the intervention with the goal of improving KT in NHs. Moreover, this investment provides unique insight into the complexity of the organization where the trial is going to take place. In turn, this study contributed invaluable information regarding the identification of needs and targets that will be articulated and addressed in the trial.

## Supplementary Information

**Additional file 1:** Interview guide

**Additional file 2:** SRQR Checklist

## Data Availability

The data that support the findings of this study are available from the Norwegian Centre for Research Data repository. Data are available upon reasonable request and with permission from the authors and the Norwegian Centre for Research Data repository (DOI: 10.18712/NSD-NSD2825-V2).

## Abbreviations

EBP: Evidence-based practice
IMPAKT: IMPlementation and Action of Knowledge Translation in practice and education
KT: Knowledge translation
MRC: Medical Research Council
NH: Nursing home
PDN: Practice development nurse
RN: Registered nurse
WHO: World Health Organization
