# Supplementary material for: Optimizing a knowledge translation intervention: a qualitative formative study to capture knowledge translation needs in nursing homes
Source: BMC Nurs. 2021 Jun 21;20:106. doi: 10.1186/s12912-021-00603-5 (PMC8218383; doi:10.1186/s12912-021-00603-5)
Supplement: Supplementary file 1 — Additional file 1: Interview guide [file 12912_2021_603_MOESM1_ESM.docx]

|  | **Interview Guide (Translated from Norwegian)** |
| --- | --- |
| 1 | (Round the table)  Can you introduce yourself, and say something about why you applied for a job as a practice development nurse? |
| 2 | Think back to when you introduced a quality improvement project, such as a new procedure, guideline or similar:   - Can you share some experiences from a successful implementation of a project - how did you experience it? - Do you have experiences with the opposite, where the implementation failed to succeed?   Potential follow up questions:  - Who decided that this project should be implemented?  - What kind of assessment of the evidence base was conducted?  - What role did you have?  - What was easy, and what was difficult? |
| 3 | - How do understand the concept of evidence-based practice (EBP)? - How would you describe your role in implementing EBP? - Who else has a role? |
| 4. | - How did you acquire knowledge about EBP? - What do you need more knowledge about? - How do you search for and where do you apply EBP? - What do you need as a practice development nurse - to be a promoter of EBP? |
| 5. | - How would you describe your position as a PDN? - How do you experience your ability to influence and change your own - and others' practice? - What do you see as positive and negative by having different roles within your position? - Do you experience anyone supporting you? - How do you experience your educational function?   - Do you have any support for this? |
| 6. | - Do you cooperate with other PDNs?   - How is it working out?  - Could it have been done differently? |
| 7. | - How is the collaboration with the management team at your nursing home regarding long-term strategy on quality work? - Do you experience yourself as a leader? Do you have authority in the context you work in? |
| 8. | - Are there other sources that influence/ triggers change?   Potential follow up questions:  Do you have any experience discussing patients and relatives views regarding professional practice in the nursing home?  Do they have the opportunity to propose changes? User surveys?  What role do you have in this regard? |
| 9. | - Is this commitment to focus on implementing EBP desirable? - Is it compatible with your priorities in your working day? - What is needed to provide you time to focus more on EBP? |
| 10. | (Round the table)   - If we were to provide a course on implementing EBP in nursing homes, what topics do you think should be addressed? |
| 11. | Repetition of the objectives of the study and summary of the discussions.   - Is this summary representative for our discussions today? - Is there something we have not talked about / something you want to add? |
| 12. | - Do you want to share any thoughts about how this focus group interview was conducted? - Any suggestions for improvement? |
